# Principles and Practice of Dentistry

**Published:** 1889-08

**Authors:** 


THE PRINCIPLES AND PRACTICE OF DENTISTRY, including Anatomy, Physiology, Pathology, Therapeutics, Dental Surgery and mechanism, by Charles A. Harris, M. D., D. D. S., Late President of the Baltimore Dental College; Author of Dictionary of Medical Terminology and Dental Surgery. Twelfth edition revised and edited by Ferdinand J. S. Gorgas, A. M., M. D., D. D. S., Author of Dental Medicine; Editor of Harris' Dictionary of Medical Terminology and Dental Surgery; Professor of the Principles of Dental Science, Dental Surgery and Dental Mechinism in the University of Maryland, with one full page plate and one thousand and twenty-eight illustrations. Philadelphia. P. Blakiston Son & Co., No 1012 Walnut St., 1889.

This is the great standard work in Dentistry, very large and comprehensive, embracing all the leading and essential points in the principles and practice of dentistry.

Twelve editions of the work have been issued with increased ability and reputation, and the present volume comes to us with two hundred and twenty-six pages of new matter and three hundred and eighty-two new illustrations, the entire volume containing twelve hundred and twenty-two large oc. pages.

The present edition was made necessary by the exhaustion of the previous one, and by the rapid advances in all departments of dental science, which in this work are fully and ably presented.

Part First of this work is devoted to anatomy and physiology in twelve parts as follows:

1st. Anatomy and physiology of the mouth.
2d. Osteology.
3d. Bones of head and face.
4th. Muscles of the mouth and face.
5th. Blood vessels of the mouth and face.
6th. Nerves of the mouth and face.
7th. Salivary glands.
8th. Tongue, gums, peridental membrane.
9th. The teeth.
10th. Malformed teeth.
11th. Origin and formation of the teeth.
12th. Osseous tooth structure.

The *Second part* contains thirteen chapters devoted to subjects connected with *pathology* and *therapeutics*. *Part third* contains ten chapters, embracing subjects in *dental surgery*.

Part fourth contains seventeen chapters devoted to dental mechanics.
Our space does not admit of further notice of this great work. Suffice it to say that no dentist who desires to be well up with his profession can afford to do without this book.

